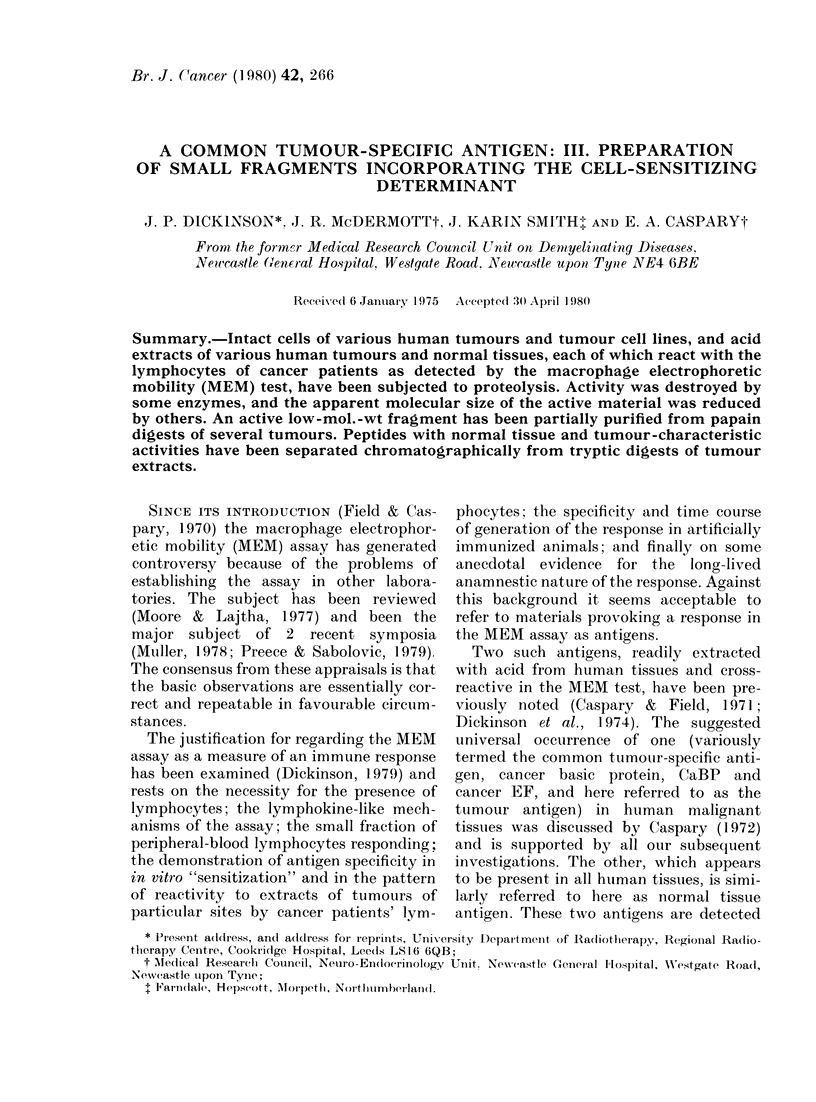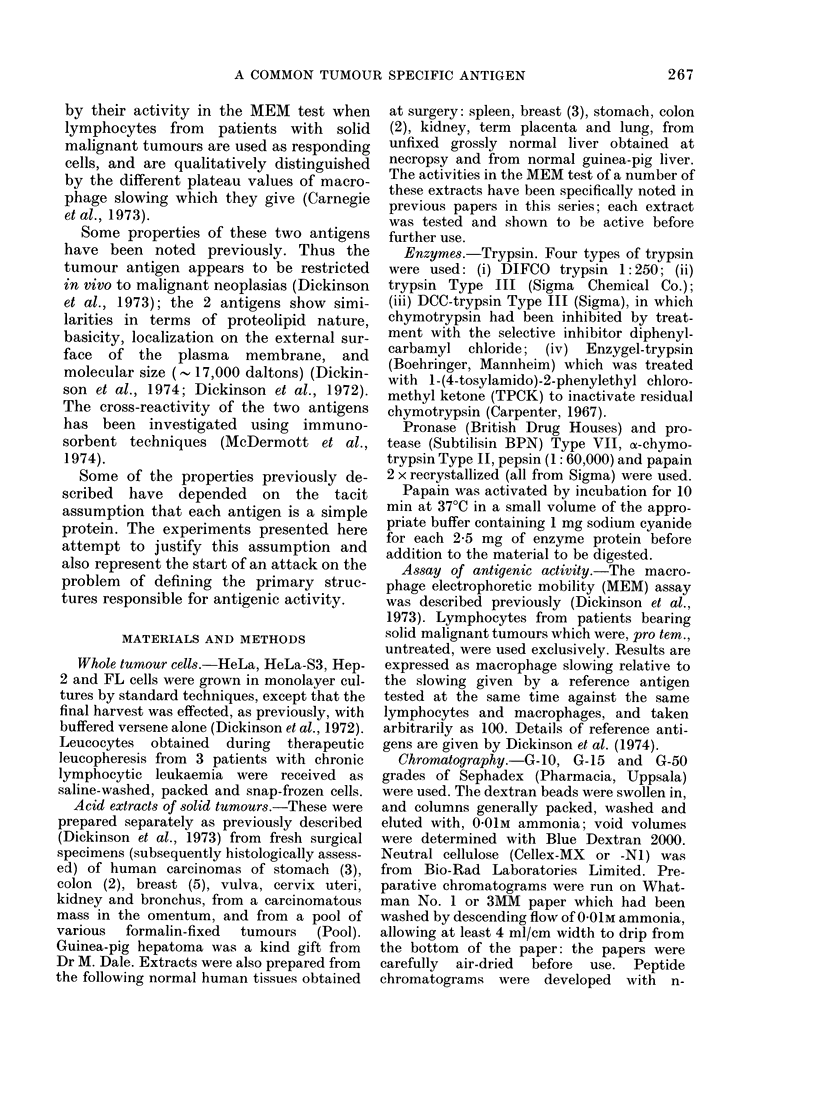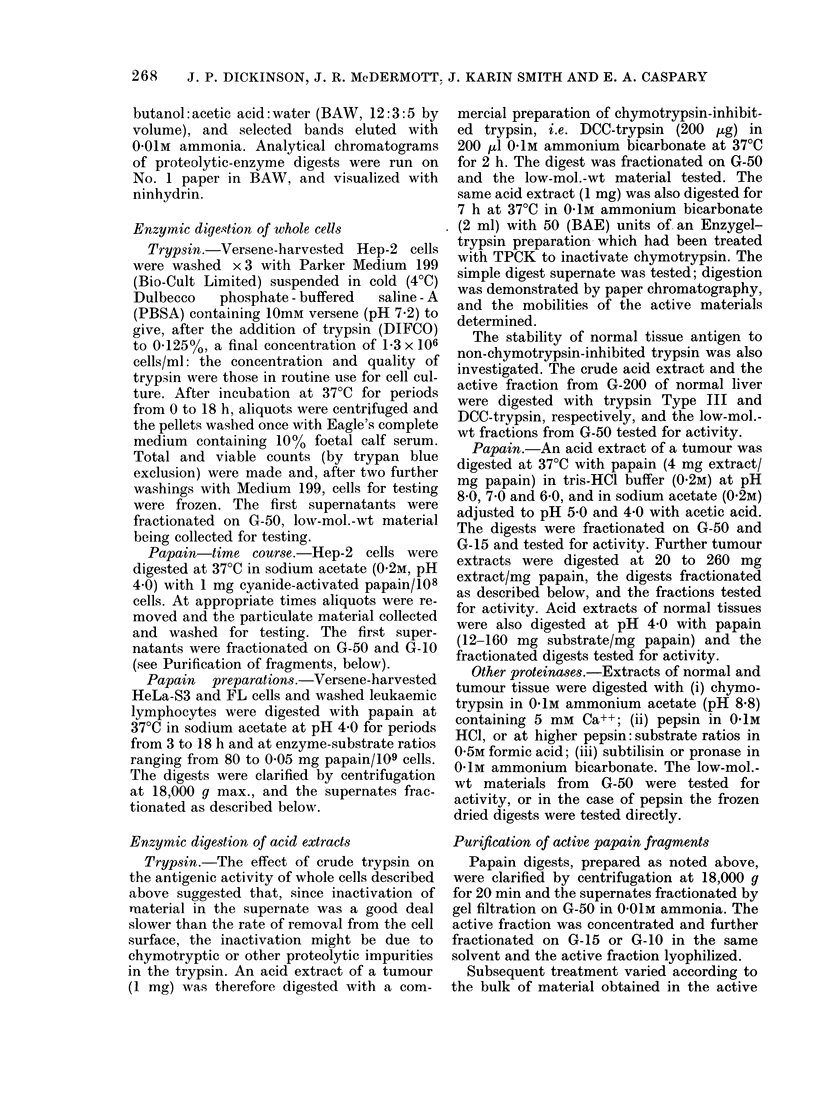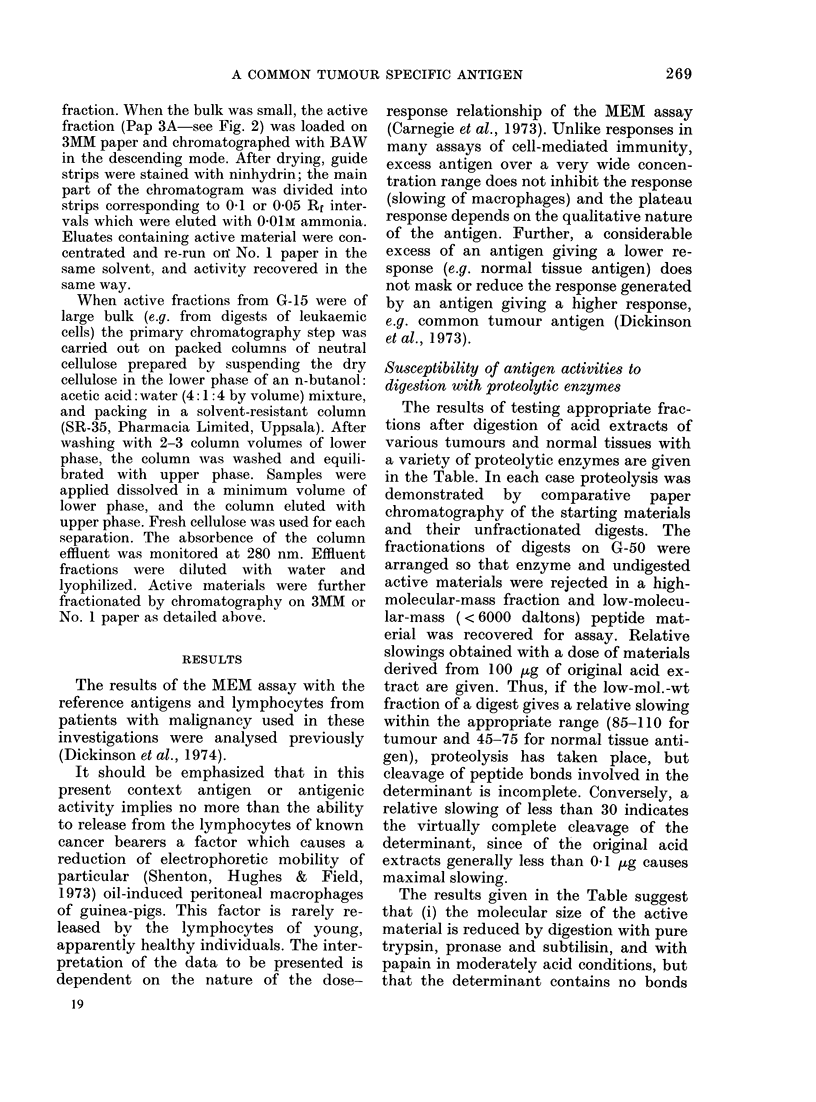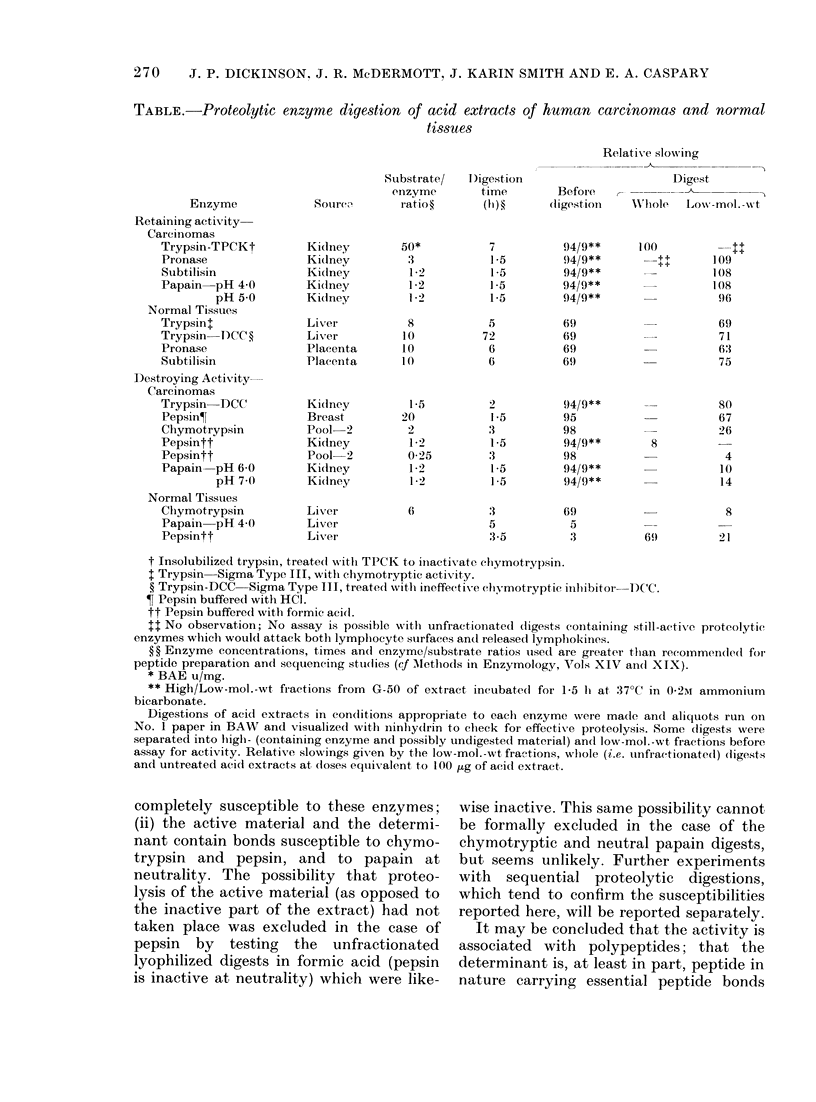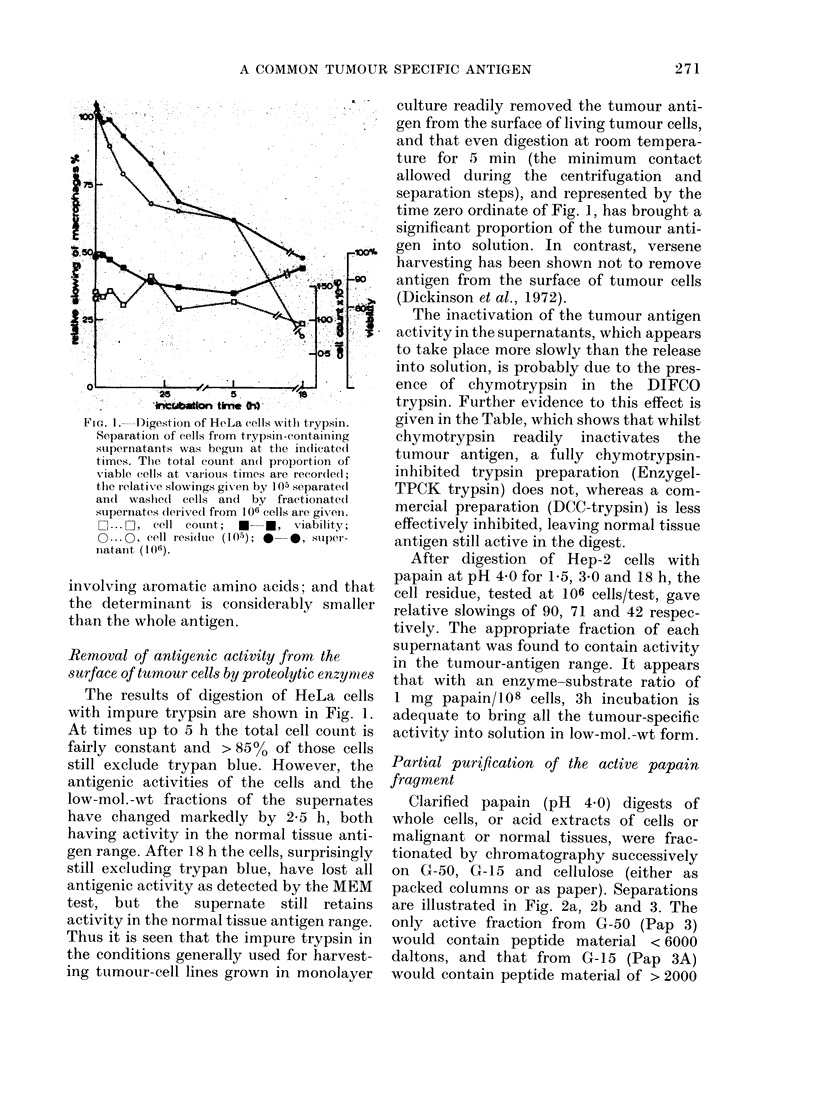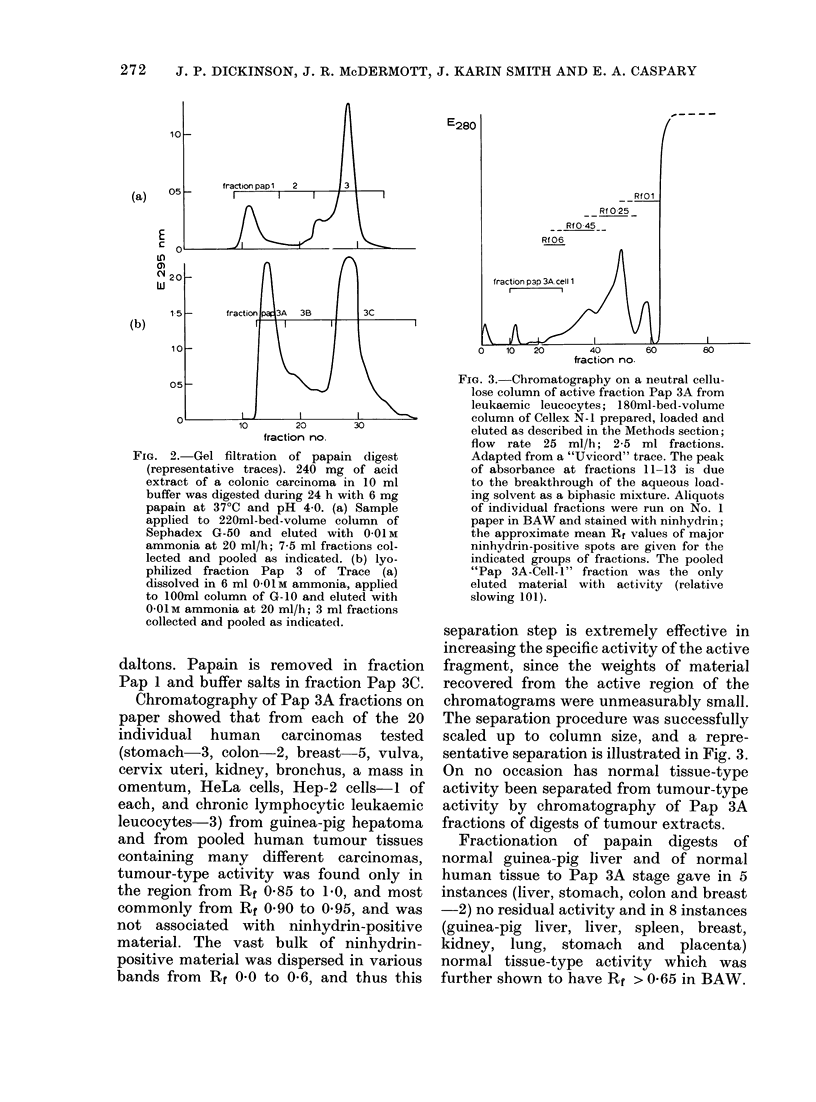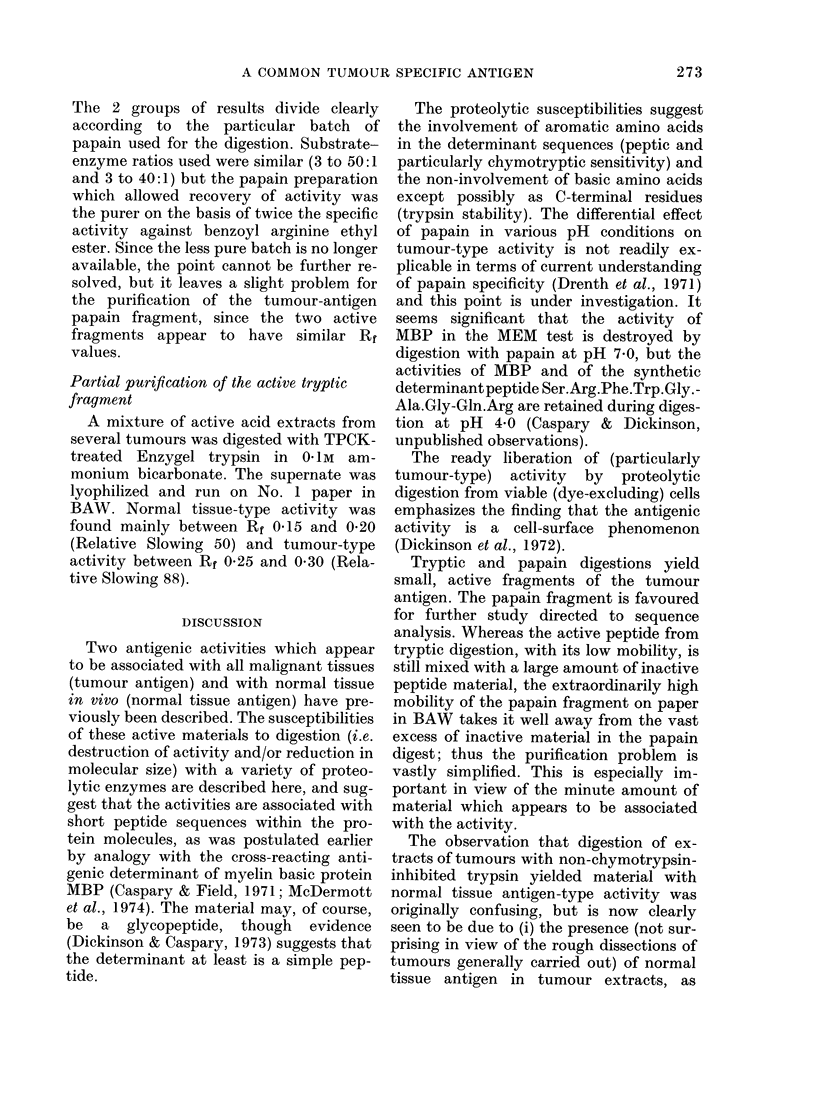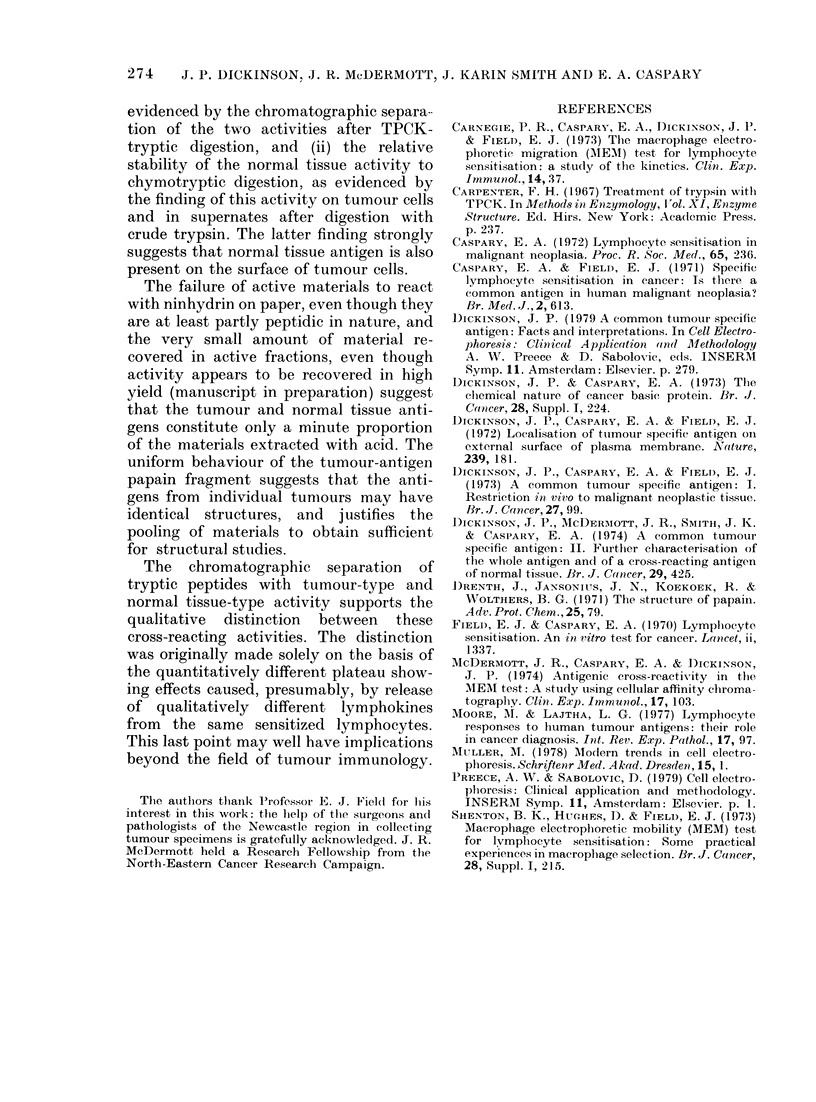# A common tumour-specific antigen: III. Preparation of small fragments incorporating the cell-sensitizing determinant.

**DOI:** 10.1038/bjc.1980.226

**Published:** 1980-08

**Authors:** J. P. Dickinson, J. R. McDermott, J. K. Smith, E. A. Caspary

## Abstract

Intact cells of various human tumours and tumour cell lines, and acid extracts of various human tumours and normal tissues, each of which react with the lymphocytes of cancer patients as detected by the macrophage electrophoretic mobility (MEM) test, have been subjected to proteolysis. Activity was destroyed by some enzymes, and the apparent molecular size of the active material was reduced by others. An active low-mol.-wt fragment has been partially purified from papain digests of several tumours. Peptides with normal tissue and tumour-characteristic activities have been separated chromatographically from tryptic digests of tumour extracts.


*Br. J. Cancer* (1980) **42**, 266

### A COMMON TUMOUR-SPECIFIC ANTIGEN: III. PREPARATION OF SMALL FRAGMENTS INCORPORATING THE CELL-SENSITIZING DETERMINANT

J. P. DICKINSON*, J. R. McDERMOTT†, J. KARIN SMITH‡ AND E. A. CASPARY†

*From the former Medical Research Council Unit on Demyelinating Diseases, Newcastle General Hospital, Westgate Road, Newcastle upon Tyne NE4 6BE*

Received 6 January 1975 Accepted 30 April 1980

**Summary.—Intact cells of various human tumours and tumour cell lines, and acid extracts of various human tumours and normal tissues, each of which react with the lymphocytes of cancer patients as detected by the macrophage electrophoretic mobility (MEM) test, have been subjected to proteolysis. Activity was destroyed by some enzymes, and the apparent molecular size of the active material was reduced by others. An active low-mol.-wt fragment has been partially purified from papain digests of several tumours. Peptides with normal tissue and tumour-characteristic activities have been separated chromatographically from tryptic digests of tumour extracts.**

SINCE ITS INTRODUCTION (Field & Caspary, 1970) the macrophage electrophoretic mobility (MEM) assay has generated controversy because of the problems of establishing the assay in other laboratories. The subject has been reviewed (Moore & Lajtha, 1977) and been the major subject of 2 recent symposia (Muller, 1978; Preece & Sabolovic, 1979). The consensus from these appraisals is that the basic observations are essentially correct and repeatable in favourable circumstances.

The justification for regarding the MEM assay as a measure of an immune response has been examined (Dickinson, 1979) and rests on the necessity for the presence of lymphocytes; the lymphokine-like mechanisms of the assay; the small fraction of peripheral-blood lymphocytes responding; the demonstration of antigen specificity in *in vitro* "sensitization" and in the pattern of reactivity to extracts of tumours of particular sites by cancer patients' lymphocytes; the specificity and time course of generation of the response in artificially immunized animals; and finally on some anecdotal evidence for the long-lived anamnestic nature of the response. Against this background it seems acceptable to refer to materials provoking a response in the MEM assay as antigens.

Two such antigens, readily extracted with acid from human tissues and cross-reactive in the MEM test, have been previously noted (Caspary & Field, 1971; Dickinson *et al.*, 1974). The suggested universal occurrence of one (variously termed the common tumour-specific antigen, cancer basic protein, CaBP and cancer EF, and here referred to as the tumour antigen) in human malignant tissues was discussed by Caspary (1972) and is supported by all our subsequent investigations. The other, which appears to be present in all human tissues, is similarly referred to here as normal tissue antigen. These two antigens are detected

* Present address, and address for reprints, University Department of Radiotherapy, Regional Radiotherapy Centre, Cookridge Hospital, Leeds LS16 6QB;

† Medical Research Council, Neuro-Endocrinology Unit, Newcastle General Hospital, Westgate Road, Newcastle upon Tyne;

‡ Farndale, Hepscott, Morpeth, Northumberland.

by their activity in the MEM test when lymphocytes from patients with solid malignant tumours are used as responding cells, and are qualitatively distinguished by the different plateau values of macrophage slowing which they give (Carnegie *et al.*, 1973).

Some properties of these two antigens have been noted previously. Thus the tumour antigen appears to be restricted *in vivo* to malignant neoplasias (Dickinson *et al.*, 1973); the 2 antigens show similarities in terms of proteolipid nature, basicity, localization on the external surface of the plasma membrane, and molecular size ($\sim$17,000 daltons) (Dickinson *et al.*, 1974; Dickinson *et al.*, 1972). The cross-reactivity of the two antigens has been investigated using immunosorbent techniques (McDermott *et al.*, 1974).

Some of the properties previously described have depended on the tacit assumption that each antigen is a simple protein. The experiments presented here attempt to justify this assumption and also represent the start of an attack on the problem of defining the primary structures responsible for antigenic activity.

### MATERIALS AND METHODS

*Whole tumour cells.*—HeLa, HeLa-S3, Hep-2 and FL cells were grown in monolayer cultures by standard techniques, except that the final harvest was effected, as previously, with buffered versene alone (Dickinson *et al.*, 1972). Leucocytes obtained during therapeutic leucopheresis from 3 patients with chronic lymphocytic leukaemia were received as saline-washed, packed and snap-frozen cells.

*Acid extracts of solid tumours.*—These were prepared separately as previously described (Dickinson *et al.*, 1973) from fresh surgical specimens (subsequently histologically assessed) of human carcinomas of stomach (3), colon (2), breast (5), vulva, cervix uteri, kidney and bronchus, from a carcinomatous mass in the omentum, and from a pool of various formalin-fixed tumours (Pool). Guinea-pig hepatoma was a kind gift from Dr M. Dale. Extracts were also prepared from the following normal human tissues obtained at surgery: spleen, breast (3), stomach, colon (2), kidney, term placenta and lung, from unfixed grossly normal liver obtained at necropsy and from normal guinea-pig liver. The activities in the MEM test of a number of these extracts have been specifically noted in previous papers in this series; each extract was tested and shown to be active before further use.

*Enzymes.*—Trypsin. Four types of trypsin were used: (i) DIFCO trypsin 1:250; (ii) trypsin Type III (Sigma Chemical Co.); (iii) DCC-trypsin Type III (Sigma), in which chymotrypsin had been inhibited by treatment with the selective inhibitor diphenylcarbamyl chloride; (iv) Enzygel-trypsin (Boehringer, Mannheim) which was treated with 1-(4-tosylamido)-2-phenylethyl chloromethyl ketone (TPCK) to inactivate residual chymotrypsin (Carpenter, 1967).

Pronase (British Drug Houses) and protease (Subtilisin BPN) Type VII, α-chymotrypsin Type II, pepsin (1:60,000) and papain 2 × recrystallized (all from Sigma) were used.

Papain was activated by incubation for 10 min at 37°C in a small volume of the appropriate buffer containing 1 mg sodium cyanide for each 2·5 mg of enzyme protein before addition to the material to be digested.

*Assay of antigenic activity.*—The macrophage electrophoretic mobility (MEM) assay was described previously (Dickinson *et al.*, 1973). Lymphocytes from patients bearing solid malignant tumours which were, *pro tem.*, untreated, were used exclusively. Results are expressed as macrophage slowing relative to the slowing given by a reference antigen tested at the same time against the same lymphocytes and macrophages, and taken arbitrarily as 100. Details of reference antigens are given by Dickinson *et al.* (1974).

*Chromatography.*—G-10, G-15 and G-50 grades of Sephadex (Pharmacia, Uppsala) were used. The dextran beads were swollen in, and columns generally packed, washed and eluted with, 0·01M ammonia; void volumes were determined with Blue Dextran 2000. Neutral cellulose (Cellex-MX or -N1) was from Bio-Rad Laboratories Limited. Preparative chromatograms were run on Whatman No. 1 or 3MM paper which had been washed by descending flow of 0·01M ammonia, allowing at least 4 ml/cm width to drip from the bottom of the paper: the papers were carefully air-dried before use. Peptide chromatograms were developed with nbutanol:acetic acid:water (BAW, 12:3:5 by volume), and selected bands eluted with 0·01M ammonia. Analytical chromatograms of proteolytic-enzyme digests were run on No. 1 paper in BAW, and visualized with ninhydrin.

*Enzymic digestion of whole cells*

*Trypsin.*—Versene-harvested Hep-2 cells were washed ×3 with Parker Medium 199 (Bio-Cult Limited) suspended in cold (4°C) Dulbecco phosphate-buffered saline-A (PBSA) containing 10mM versene (pH 7·2) to give, after the addition of trypsin (DIFCO) to 0·125%, a final concentration of $1{\cdot}3 \times 10^6$ cells/ml: the concentration and quality of trypsin were those in routine use for cell culture. After incubation at 37°C for periods from 0 to 18 h, aliquots were centrifuged and the pellets washed once with Eagle's complete medium containing 10% foetal calf serum. Total and viable counts (by trypan blue exclusion) were made and, after two further washings with Medium 199, cells for testing were frozen. The first supernatants were fractionated on G-50, low-mol.-wt material being collected for testing.

*Papain—time course.*—Hep-2 cells were digested at 37°C in sodium acetate (0·2M, pH 4·0) with 1 mg cyanide-activated papain/$10^8$ cells. At appropriate times aliquots were removed and the particulate material collected and washed for testing. The first supernatants were fractionated on G-50 and G-10 (see Purification of fragments, below).

*Papain preparations.*—Versene-harvested HeLa-S3 and FL cells and washed leukaemic lymphocytes were digested with papain at 37°C in sodium acetate at pH 4·0 for periods from 3 to 18 h and at enzyme-substrate ratios ranging from 80 to 0·05 mg papain/$10^9$ cells. The digests were clarified by centrifugation at 18,000 *g* max., and the supernates fractionated as described below.

*Enzymic digestion of acid extracts*

*Trypsin.*—The effect of crude trypsin on the antigenic activity of whole cells described above suggested that, since inactivation of material in the supernate was a good deal slower than the rate of removal from the cell surface, the inactivation might be due to chymotryptic or other proteolytic impurities in the trypsin. An acid extract of a tumour (1 mg) was therefore digested with a commercial preparation of chymotrypsin-inhibited trypsin, *i.e.* DCC-trypsin (200 μg) in 200 μl 0·1M ammonium bicarbonate at 37°C for 2 h. The digest was fractionated on G-50 and the low-mol.-wt material tested. The same acid extract (1 mg) was also digested for 7 h at 37°C in 0·1M ammonium bicarbonate (2 ml) with 50 (BAE) units of an Enzygel-trypsin preparation which had been treated with TPCK to inactivate chymotrypsin. The simple digest supernate was tested; digestion was demonstrated by paper chromatography, and the mobilities of the active materials determined.

The stability of normal tissue antigen to non-chymotrypsin-inhibited trypsin was also investigated. The crude acid extract and the active fraction from G-200 of normal liver were digested with trypsin Type III and DCC-trypsin, respectively, and the low-mol.-wt fractions from G-50 tested for activity.

*Papain.*—An acid extract of a tumour was digested at 37°C with papain (4 mg extract/mg papain) in tris-HCl buffer (0·2M) at pH 8·0, 7·0 and 6·0, and in sodium acetate (0·2M) adjusted to pH 5·0 and 4·0 with acetic acid. The digests were fractionated on G-50 and G-15 and tested for activity. Further tumour extracts were digested at 20 to 260 mg extract/mg papain, the digests fractionated as described below, and the fractions tested for activity. Acid extracts of normal tissues were also digested at pH 4·0 with papain (12–160 mg substrate/mg papain) and the fractionated digests tested for activity.

*Other proteinases.*—Extracts of normal and tumour tissue were digested with (i) chymotrypsin in 0·1M ammonium acetate (pH 8·8) containing 5 mM $Ca^{++}$; (ii) pepsin in 0·1M HCl, or at higher pepsin:substrate ratios in 0·5M formic acid; (iii) subtilisin or pronase in 0·1M ammonium bicarbonate. The low-mol.-wt materials from G-50 were tested for activity, or in the case of pepsin the frozen dried digests were tested directly.

*Purification of active papain fragments*

Papain digests, prepared as noted above, were clarified by centrifugation at 18,000 *g* for 20 min and the supernates fractionated by gel filtration on G-50 in 0·01M ammonia. The active fraction was concentrated and further fractionated on G-15 or G-10 in the same solvent and the active fraction lyophilized.

Subsequent treatment varied according to the bulk of material obtained in the active fraction. When the bulk was small, the active fraction (Pap 3A—see Fig. 2) was loaded on 3MM paper and chromatographed with BAW in the descending mode. After drying, guide strips were stained with ninhydrin; the main part of the chromatogram was divided into strips corresponding to 0·1 or 0·05 $R_f$ intervals which were eluted with 0·01M ammonia. Eluates containing active material were concentrated and re-run on No. 1 paper in the same solvent, and activity recovered in the same way.

When active fractions from G-15 were of large bulk (*e.g.* from digests of leukaemic cells) the primary chromatography step was carried out on packed columns of neutral cellulose prepared by suspending the dry cellulose in the lower phase of an n-butanol: acetic acid: water (4:1:4 by volume) mixture, and packing in a solvent-resistant column (SR-35, Pharmacia Limited, Uppsala). After washing with 2–3 column volumes of lower phase, the column was washed and equilibrated with upper phase. Samples were applied dissolved in a minimum volume of lower phase, and the column eluted with upper phase. Fresh cellulose was used for each separation. The absorbence of the column effluent was monitored at 280 nm. Effluent fractions were diluted with water and lyophilized. Active materials were further fractionated by chromatography on 3MM or No. 1 paper as detailed above.

### RESULTS

The results of the MEM assay with the reference antigens and lymphocytes from patients with malignancy used in these investigations were analysed previously (Dickinson *et al.*, 1974).

It should be emphasized that in this present context antigen or antigenic activity implies no more than the ability to release from the lymphocytes of known cancer bearers a factor which causes a reduction of electrophoretic mobility of particular (Shenton, Hughes & Field, 1973) oil-induced peritoneal macrophages of guinea-pigs. This factor is rarely released by the lymphocytes of young, apparently healthy individuals. The interpretation of the data to be presented is dependent on the nature of the dose–response relationship of the MEM assay (Carnegie *et al.*, 1973). Unlike responses in many assays of cell-mediated immunity, excess antigen over a very wide concentration range does not inhibit the response (slowing of macrophages) and the plateau response depends on the qualitative nature of the antigen. Further, a considerable excess of an antigen giving a lower response (*e.g.* normal tissue antigen) does not mask or reduce the response generated by an antigen giving a higher response, *e.g.* common tumour antigen (Dickinson *et al.*, 1973).

### *Susceptibility of antigen activities to digestion with proteolytic enzymes*

The results of testing appropriate fractions after digestion of acid extracts of various tumours and normal tissues with a variety of proteolytic enzymes are given in the Table. In each case proteolysis was demonstrated by comparative paper chromatography of the starting materials and their unfractionated digests. The fractionations of digests on G-50 were arranged so that enzyme and undigested active materials were rejected in a high-molecular-mass fraction and low-molecular-mass (< 6000 daltons) peptide material was recovered for assay. Relative slowings obtained with a dose of materials derived from 100 $\mu$g of original acid extract are given. Thus, if the low-mol.-wt fraction of a digest gives a relative slowing within the appropriate range (85–110 for tumour and 45–75 for normal tissue antigen), proteolysis has taken place, but cleavage of peptide bonds involved in the determinant is incomplete. Conversely, a relative slowing of less than 30 indicates the virtually complete cleavage of the determinant, since of the original acid extracts generally less than 0·1 $\mu$g causes maximal slowing.

The results given in the Table suggest that (i) the molecular size of the active material is reduced by digestion with pure trypsin, pronase and subtilisin, and with papain in moderately acid conditions, but that the determinant contains no bonds

TABLE.—*Proteolytic enzyme digestion of acid extracts of human carcinomas and normal tissues*

| | | | | Relative slowing | | |
|---|---|---|---|---|---|---|
| | | | | | Digest | |
| Enzyme | Source | Substrate/ enzyme ratio§ | Digestion time (h)§ | Before digestion | Whole | Low-mol.-wt |
| Retaining activity— | | | | | | |
| Carcinomas | | | | | | |
| Trypsin-TPCK† | Kidney | 50* | 7 | 94/9** | 100 | —‡‡ |
| Pronase | Kidney | 3 | 1·5 | 94/9** | —‡‡ | 109 |
| Subtilisin | Kidney | 1·2 | 1·5 | 94/9** | — | 108 |
| Papain—pH 4·0 | Kidney | 1·2 | 1·5 | 94/9** | — | 108 |
| pH 5·0 | Kidney | 1·2 | 1·5 | 94/9** | — | 96 |
| Normal Tissues | | | | | | |
| Trypsin‡ | Liver | 8 | 5 | 69 | — | 69 |
| Trypsin—DCC§ | Liver | 10 | 72 | 69 | — | 71 |
| Pronase | Placenta | 10 | 6 | 69 | — | 63 |
| Subtilisin | Placenta | 10 | 6 | 69 | — | 75 |
| Destroying Activity— | | | | | | |
| Carcinomas | | | | | | |
| Trypsin—DCC | Kidney | 1·5 | 2 | 94/9** | — | 80 |
| Pepsin¶ | Breast | 20 | 1·5 | 95 | — | 67 |
| Chymotrypsin | Pool—2 | 2 | 3 | 98 | — | 26 |
| Pepsin†† | Kidney | 1·2 | 1·5 | 94/9** | 8 | — |
| Pepsin†† | Pool—2 | 0·25 | 3 | 98 | — | 4 |
| Papain—pH 6·0 | Kidney | 1·2 | 1·5 | 94/9** | — | 10 |
| pH 7·0 | Kidney | 1·2 | 1·5 | 94/9** | — | 14 |
| Normal Tissues | | | | | | |
| Chymotrypsin | Liver | 6 | 3 | 69 | — | 8 |
| Papain—pH 4·0 | Liver | | 5 | 5 | — | — |
| Pepsin†† | Liver | | 3·5 | 3 | 69 | 21 |

† Insolubilized trypsin, treated with TPCK to inactivate chymotrypsin.
‡ Trypsin—Sigma Type III, with chymotryptic activity.
§ Trypsin-DCC—Sigma Type III, treated with ineffective chymotryptic inhibitor—DCC.
¶ Pepsin buffered with HCl.
†† Pepsin buffered with formic acid.
‡‡ No observation; No assay is possible with unfractionated digests containing still-active proteolytic enzymes which would attack both lymphocyte surfaces and released lymphokines.
§§ Enzyme concentrations, times and enzyme/substrate ratios used are greater than recommended for peptide preparation and sequencing studies (*cf* Methods in Enzymology, Vols XIV and XIX).
* BAE u/mg.
** High/Low-mol.-wt fractions from G-50 of extract incubated for 1·5 h at 37°C in 0·2M ammonium bicarbonate.

Digestions of acid extracts in conditions appropriate to each enzyme were made and aliquots run on No. 1 paper in BAW and visualized with ninhydrin to check for effective proteolysis. Some digests were separated into high- (containing enzyme and possibly undigested material) and low-mol.-wt fractions before assay for activity. Relative slowings given by the low-mol.-wt fractions, whole (*i.e.* unfractionated) digests and untreated acid extracts at doses equivalent to 100 μg of acid extract.

completely susceptible to these enzymes; (ii) the active material and the determinant contain bonds susceptible to chymotrypsin and pepsin, and to papain at neutrality. The possibility that proteolysis of the active material (as opposed to the inactive part of the extract) had not taken place was excluded in the case of pepsin by testing the unfractionated lyophilized digests in formic acid (pepsin is inactive at neutrality) which were likewise inactive. This same possibility cannot be formally excluded in the case of the chymotryptic and neutral papain digests, but seems unlikely. Further experiments with sequential proteolytic digestions, which tend to confirm the susceptibilities reported here, will be reported separately.

It may be concluded that the activity is associated with polypeptides; that the determinant is, at least in part, peptide in nature carrying essential peptide bonds

FIG. 1.—Digestion of HeLa cells with trypsin. Separation of cells from trypsin-containing supernatants was begun at the indicated times. The total count and proportion of viable cells at various times are recorded; the relative slowings given by $10^5$ separated and washed cells and by fractionated supernates derived from $10^6$ cells are given. □...□, cell count; ■—■, viability; ○...○, cell residue ($10^5$); ●—●, supernatant ($10^6$).

involving aromatic amino acids; and that the determinant is considerably smaller than the whole antigen.

*Removal of antigenic activity from the surface of tumour cells by proteolytic enzymes*

The results of digestion of HeLa cells with impure trypsin are shown in Fig. 1. At times up to 5 h the total cell count is fairly constant and >85% of those cells still exclude trypan blue. However, the antigenic activities of the cells and the low-mol.-wt fractions of the supernates have changed markedly by 2·5 h, both having activity in the normal tissue antigen range. After 18 h the cells, surprisingly still excluding trypan blue, have lost all antigenic activity as detected by the MEM test, but the supernate still retains activity in the normal tissue antigen range. Thus it is seen that the impure trypsin in the conditions generally used for harvesting tumour-cell lines grown in monolayer culture readily removed the tumour antigen from the surface of living tumour cells, and that even digestion at room temperature for 5 min (the minimum contact allowed during the centrifugation and separation steps), and represented by the time zero ordinate of Fig. 1, has brought a significant proportion of the tumour antigen into solution. In contrast, versene harvesting has been shown not to remove antigen from the surface of tumour cells (Dickinson *et al.*, 1972).

The inactivation of the tumour antigen activity in the supernatants, which appears to take place more slowly than the release into solution, is probably due to the presence of chymotrypsin in the DIFCO trypsin. Further evidence to this effect is given in the Table, which shows that whilst chymotrypsin readily inactivates the tumour antigen, a fully chymotrypsin-inhibited trypsin preparation (Enzygel-TPCK trypsin) does not, whereas a commercial preparation (DCC-trypsin) is less effectively inhibited, leaving normal tissue antigen still active in the digest.

After digestion of Hep-2 cells with papain at pH 4·0 for 1·5, 3·0 and 18 h, the cell residue, tested at $10^6$ cells/test, gave relative slowings of 90, 71 and 42 respectively. The appropriate fraction of each supernatant was found to contain activity in the tumour-antigen range. It appears that with an enzyme–substrate ratio of 1 mg papain/$10^8$ cells, 3h incubation is adequate to bring all the tumour-specific activity into solution in low-mol.-wt form.

*Partial purification of the active papain fragment*

Clarified papain (pH 4·0) digests of whole cells, or acid extracts of cells or malignant or normal tissues, were fractionated by chromatography successively on G-50, G-15 and cellulose (either as packed columns or as paper). Separations are illustrated in Fig. 2a, 2b and 3. The only active fraction from G-50 (Pap 3) would contain peptide material <6000 daltons, and that from G-15 (Pap 3A) would contain peptide material of >2000

FIG. 2.—Gel filtration of papain digest (representative traces). 240 mg of acid extract of a colonic carcinoma in 10 ml buffer was digested during 24 h with 6 mg papain at 37°C and pH 4·0. (a) Sample applied to 220ml-bed-volume column of Sephadex G-50 and eluted with 0·01 M ammonia at 20 ml/h; 7·5 ml fractions collected and pooled as indicated. (b) lyophilized fraction Pap 3 of Trace (a) dissolved in 6 ml 0·01 M ammonia, applied to 100ml column of G-10 and eluted with 0·01 M ammonia at 20 ml/h; 3 ml fractions collected and pooled as indicated.

FIG. 3.—Chromatography on a neutral cellulose column of active fraction Pap 3A from leukaemic leucocytes; 180ml-bed-volume column of Cellex N-1 prepared, loaded and eluted as described in the Methods section; flow rate 25 ml/h; 2·5 ml fractions. Adapted from a "Uvicord" trace. The peak of absorbance at fractions 11–13 is due to the breakthrough of the aqueous loading solvent as a biphasic mixture. Aliquots of individual fractions were run on No. 1 paper in BAW and stained with ninhydrin; the approximate mean $R_f$ values of major ninhydrin-positive spots are given for the indicated groups of fractions. The pooled "Pap 3A-Cell-1" fraction was the only eluted material with activity (relative slowing 101).

daltons. Papain is removed in fraction Pap 1 and buffer salts in fraction Pap 3C.

Chromatography of Pap 3A fractions on paper showed that from each of the 20 individual human carcinomas tested (stomach—3, colon—2, breast—5, vulva, cervix uteri, kidney, bronchus, a mass in omentum, HeLa cells, Hep-2 cells—1 of each, and chronic lymphocytic leukaemic leucocytes—3) from guinea-pig hepatoma and from pooled human tumour tissues containing many different carcinomas, tumour-type activity was found only in the region from $R_f$ 0·85 to 1·0, and most commonly from $R_f$ 0·90 to 0·95, and was not associated with ninhydrin-positive material. The vast bulk of ninhydrin-positive material was dispersed in various bands from $R_f$ 0·0 to 0·6, and thus this separation step is extremely effective in increasing the specific activity of the active fragment, since the weights of material recovered from the active region of the chromatograms were unmeasurably small. The separation procedure was successfully scaled up to column size, and a representative separation is illustrated in Fig. 3. On no occasion has normal tissue-type activity been separated from tumour-type activity by chromatography of Pap 3A fractions of digests of tumour extracts.

Fractionation of papain digests of normal guinea-pig liver and of normal human tissue to Pap 3A stage gave in 5 instances (liver, stomach, colon and breast —2) no residual activity and in 8 instances (guinea-pig liver, liver, spleen, breast, kidney, lung, stomach and placenta) normal tissue-type activity which was further shown to have $R_f$ > 0·65 in BAW.

The 2 groups of results divide clearly according to the particular batch of papain used for the digestion. Substrate–enzyme ratios used were similar (3 to 50:1 and 3 to 40:1) but the papain preparation which allowed recovery of activity was the purer on the basis of twice the specific activity against benzoyl arginine ethyl ester. Since the less pure batch is no longer available, the point cannot be further resolved, but it leaves a slight problem for the purification of the tumour-antigen papain fragment, since the two active fragments appear to have similar $R_f$ values.

*Partial purification of the active tryptic fragment*

A mixture of active acid extracts from several tumours was digested with TPCK-treated Enzygel trypsin in 0·1M ammonium bicarbonate. The supernate was lyophilized and run on No. 1 paper in BAW. Normal tissue-type activity was found mainly between $R_f$ 0·15 and 0·20 (Relative Slowing 50) and tumour-type activity between $R_f$ 0·25 and 0·30 (Relative Slowing 88).

### DISCUSSION

Two antigenic activities which appear to be associated with all malignant tissues (tumour antigen) and with normal tissue *in vivo* (normal tissue antigen) have previously been described. The susceptibilities of these active materials to digestion (*i.e.* destruction of activity and/or reduction in molecular size) with a variety of proteolytic enzymes are described here, and suggest that the activities are associated with short peptide sequences within the protein molecules, as was postulated earlier by analogy with the cross-reacting antigenic determinant of myelin basic protein MBP (Caspary & Field, 1971; McDermott *et al.*, 1974). The material may, of course, be a glycopeptide, though evidence (Dickinson & Caspary, 1973) suggests that the determinant at least is a simple peptide.

The proteolytic susceptibilities suggest the involvement of aromatic amino acids in the determinant sequences (peptic and particularly chymotryptic sensitivity) and the non-involvement of basic amino acids except possibly as C-terminal residues (trypsin stability). The differential effect of papain in various pH conditions on tumour-type activity is not readily explicable in terms of current understanding of papain specificity (Drenth *et al.*, 1971) and this point is under investigation. It seems significant that the activity of MBP in the MEM test is destroyed by digestion with papain at pH 7·0, but the activities of MBP and of the synthetic determinant peptide Ser.Arg.Phe.Trp.Gly.-Ala.Gly-Gln.Arg are retained during digestion at pH 4·0 (Caspary & Dickinson, unpublished observations).

The ready liberation of (particularly tumour-type) activity by proteolytic digestion from viable (dye-excluding) cells emphasizes the finding that the antigenic activity is a cell-surface phenomenon (Dickinson *et al.*, 1972).

Tryptic and papain digestions yield small, active fragments of the tumour antigen. The papain fragment is favoured for further study directed to sequence analysis. Whereas the active peptide from tryptic digestion, with its low mobility, is still mixed with a large amount of inactive peptide material, the extraordinarily high mobility of the papain fragment on paper in BAW takes it well away from the vast excess of inactive material in the papain digest; thus the purification problem is vastly simplified. This is especially important in view of the minute amount of material which appears to be associated with the activity.

The observation that digestion of extracts of tumours with non-chymotrypsin-inhibited trypsin yielded material with normal tissue antigen-type activity was originally confusing, but is now clearly seen to be due to (i) the presence (not surprising in view of the rough dissections of tumours generally carried out) of normal tissue antigen in tumour extracts, as evidenced by the chromatographic separation of the two activities after TPCK-tryptic digestion, and (ii) the relative stability of the normal tissue activity to chymotryptic digestion, as evidenced by the finding of this activity on tumour cells and in supernates after digestion with crude trypsin. The latter finding strongly suggests that normal tissue antigen is also present on the surface of tumour cells.

The failure of active materials to react with ninhydrin on paper, even though they are at least partly peptidic in nature, and the very small amount of material recovered in active fractions, even though activity appears to be recovered in high yield (manuscript in preparation) suggest that the tumour and normal tissue antigens constitute only a minute proportion of the materials extracted with acid. The uniform behaviour of the tumour-antigen papain fragment suggests that the antigens from individual tumours may have identical structures, and justifies the pooling of materials to obtain sufficient for structural studies.

The chromatographic separation of tryptic peptides with tumour-type and normal tissue-type activity supports the qualitative distinction between these cross-reacting activities. The distinction was originally made solely on the basis of the quantitatively different plateau showing effects caused, presumably, by release of qualitatively different lymphokines from the same sensitized lymphocytes. This last point may well have implications beyond the field of tumour immunology.

The authors thank Professor E. J. Field for his interest in this work: the help of the surgeons and pathologists of the Newcastle region in collecting tumour specimens is gratefully acknowledged. J. R. McDermott held a Research Fellowship from the North-Eastern Cancer Research Campaign.

### REFERENCES

CARNEGIE, P. R., CASPARY, E. A., DICKINSON, J. P. & FIELD, E. J. (1973) The macrophage electrophoretic migration (MEM) test for lymphocyte sensitisation: a study of the kinetics. *Clin. Exp. Immunol.*, **14**, 37.

CARPENTER, F. H. (1967) Treatment of trypsin with TPCK. In *Methods in Enzymology, Vol. XI, Enzyme Structure*. Ed. Hirs. New York: Academic Press. p. 237.

CASPARY, E. A. (1972) Lymphocyte sensitisation in malignant neoplasia. *Proc. R. Soc. Med.*, **65**, 236.

CASPARY, E. A. & FIELD, E. J. (1971) Specific lymphocyte sensitisation in cancer: Is there a common antigen in human malignant neoplasia? *Br. Med. J.*, **2**, 613.

DICKINSON, J. P. (1979 A common tumour specific antigen: Facts and interpretations. In *Cell Electrophoresis: Clinical Application and Methodology* A. W. Preece & D. Sabolovic, eds. INSERM Symp. **11**. Amsterdam: Elsevier. p. 279.

DICKINSON, J. P. & CASPARY, E. A. (1973) The chemical nature of cancer basic protein. *Br. J. Cancer*, **28**, Suppl. I, 224.

DICKINSON, J. P., CASPARY, E. A. & FIELD, E. J. (1972) Localisation of tumour specific antigen on external surface of plasma membrane. *Nature*, **239**, 181.

DICKINSON, J. P., CASPARY, E. A. & FIELD, E. J. (1973) A common tumour specific antigen: I. Restriction *in vivo* to malignant neoplastic tissue. *Br. J. Cancer*, **27**, 99.

DICKINSON, J. P., McDERMOTT, J. R., SMITH, J. K. & CASPARY, E. A. (1974) A common tumour specific antigen: II. Further characterisation of the whole antigen and of a cross-reacting antigen of normal tissue. *Br. J. Cancer*, **29**, 425.

DRENTH, J., JANSONIUS, J. N., KOEKOEK, R. & WOLTHERS, B. G. (1971) The structure of papain. *Adv. Prot. Chem.*, **25**, 79.

FIELD, E. J. & CASPARY, E. A. (1970) Lymphocyte sensitisation. An *in vitro* test for cancer. *Lancet*, ii, 1337.

McDERMOTT, J. R., CASPARY, E. A. & DICKINSON, J. P. (1974) Antigenic cross-reactivity in the MEM test: A study using cellular affinity chromatography. *Clin. Exp. Immunol.*, **17**, 103.

MOORE, M. & LAJTHA, L. G. (1977) Lymphocyte responses to human tumour antigens: their role in cancer diagnosis. *Int. Rev. Exp. Pathol.*, **17**, 97.

MULLER, M. (1978) Modern trends in cell electrophoresis. *Schriftenr Med. Akad. Dresden*, **15**, 1.

PREECE, A. W. & SABOLOVIC, D. (1979) Cell electrophoresis: Clinical application and methodology. INSERM Symp. **11**, Amsterdam: Elsevier. p. 1.

SHENTON, B. K., HUGHES, D. & FIELD, E. J. (1973) Macrophage electrophoretic mobility (MEM) test for lymphocyte sensitisation: Some practical experiences in macrophage selection. *Br. J. Cancer*, **28**, Suppl. I, 215.